# Mapping temporal trends of pesticide use and identifying potential non-occupation population exposure using a geospatial approach

**DOI:** 10.1038/s41598-025-06955-1

**Published:** 2025-07-02

**Authors:** Elisa Jazan, Timothy Griffin, Mark Woodin

**Affiliations:** 1 Department of Civil and Environmental Engineering, Tufts University School of Engineering, 200 College Ave, Medford, MA 02155 USA; 2 Friedman School of Nutrition Science and Policy, Tufts University, 150 Harrison Avenue, Boston, MA 02111 USA

**Keywords:** Pesticides, Herbicides, Agriculture, Non-occupational exposure, GIS, 2,4-D, Environmental monitoring, Environmental sciences

## Abstract

Estimating pesticide exposure in environmental settings is complex due to limited data, evolving agricultural practices, and increasing use driven by weed resistance and genetically engineered crops. One of the most widely used herbicides, 2,4-D, is a concern for populations near agricultural fields due to its rapidly increasing use. We used a geospatial approach to show spatio-temporal trends of pesticide use and identify at-risk populations for non-occupational pesticide exposure. 2,4-D application on soybeans in Illinois during the years 2017, 2020 and 2023 were used. We calculated the rate of change on the county level using reported 2,4-D use and soybean crop area. Then we created a crop area, pesticide density buffer model using 1 km buffer zones correlated with 1000 m x 1000 m gridded census data to identify where populations were at risk of non-occupational exposure in Champaign County. Between 2017 and 2023, there was a median increase of 341% in 2,4-D application on soybeans in each county in Illinois. We found that 98.9–99.7% of the population of Champaign County lived within 1 km of at least 0.04 km^2^ (10 acres) of soybean crops from 2017 to 2023. Using 4.4 kg of 2,4-D as a threshold within the 1 km buffer zone, 24.5% of the population was potentially exposed in 2017, this increased to 44.5% of the population in 2023. In 2017, the area where the most 2,4-D (30 kg) was applied to soybean crops represented 0.01% (14 people) of the population potentially exposed. Using 30 kg as a reference point, in 2023, 20.2% (~ 47,000 people) of the population were at potential risk of this level of exposure. Previous research has shown increasing 2,4-D agricultural use has been associated with increased 2,4-D physiological levels. We mapped at-risk populations for 2,4-D exposure due to 2,4-D application on soybeans that has increased from 2017 to 2023 using GIS. This is a cost-effective method that identifies areas to focus further studies.

## Introduction

Estimating potential pesticide exposure in the environmental setting is challenging, and data are limited^1,2^. Additionally, how pesticides are used is not static and modern agriculture is changing rapidly due to weed resistance and novel genetically engineered crops. Studies show that people living in close proximity to agricultural fields can experience higher pesticide exposure than those in non-agricultural areas^3–5^. Identifying at-risk populations allows for targeted resources examining the health impacts of agricultural pesticide exposure.

The herbicide, 2,4-dichlorophenoxyacetic acid (2,4-D), is one of the most commonly used herbicides globally in agriculture, forestry, and residential lawn care. In the United States (US), 2,4-D is the most common herbicide ingredient used residentially and is the second most commonly used herbicide in the agricultural sector^6^. It is a synthetic auxin herbicide that targets broadleaf plants. Introduced in the 1940s, 2,4-D was the first herbicide to be commercially available in the US. Despite 2,4-D’s long history, there is a gap in data regarding assessment of exposure^7^, and specifically non-occupational exposure^8^.

The U.S., as the world’s largest soybean producer, has seen a consistent increase in soybean production over the past 60 years. Approximately 99% of all soybean crops are treated with herbicides, with 94% of soybeans now genetically engineered (GE) to be herbicide-resistant^9^. Due to weed resistance, which herbicides are used and how they are being applied is changing.

Off target movement of pesticides, or drift, allows pesticides to make its way into homes and collect in dust and resist degradation^10^. 2,4-D has well-known issues with drift and volatility^11^. In a study of 11 states, it was found that the highest rate of pesticide poisoning among agricultural workers and residents in agricultural regions is due to drift exposure, specifically 26/643 drift events were attributed to chlorophenoxy herbicides that include 2,4-D^12^. Those who live near areas of heavy agricultural 2,4-D use often have increased exposure from dermal contact, inhalation of soil particles, and contact with people, clothing, or pets that have been exposed^13^.

2,4-D is rapidly absorbed via ingestion and inhalation routes^13^. This represents a potentially significant route of exposure, especially for children. Studies have found ingestion of dust as a primary route of exposure for children^14,15^.

Additionally, increased nearby crop area has been associated with higher concentrations of pesticides found in homes^5^.

The toxicological profile for 2,4-D indicates that it is highly mobile in soil and has the potential to migrate into groundwater^13^, also a concern for households near high-application areas.

Geographical informational systems (GIS) can integrate multiple spatial data sources to estimate potential exposure to specific pesticides in areas of interest based on proximity and location.

One of the first studies to correlate pesticide data with census data predicted population exposure to atrazine in Nebraska by using buffers around population centroids^4^. A previous study used a crop area weighted metric within buffers around residences to estimate potential exposure risk by analyzing the dust in homes^5^. The authors expanded their study design recently by incorporating pesticide density, or the intensity of application per unit area (kg/km^2^), into their research, which included 2,4-D^16^. More recent research has upscaled a similar methodology to a nationwide scale in Canada^17^, however geographical data showing changes over time are sparse. By integrating elements from each of these methods, we can show the changing use of 2,4-D correlated with population.

In this context, this study uses a geospatial approach to estimate potential pesticide exposure due to agricultural use using crop, pesticide, and census data, while also examining the recent temporal trends of pesticide use. By focusing on 2,4-dichlorophenoxyacetic acid (2,4-D) herbicide applications on soybean crops in Illinois as a case study, this paper aims to map and assess non-occupational potential exposure risks for nearby populations and how it is changing over time.

## Methods

### Study area

The years 2017, 2020 and 2023 were used to examine the increasing use of 2,4-D based on data availability and 2,4-D resistant soybeans becoming widely available commercially in 2019^18^.

The state of Illinois was selected as the study area, as it is the top producer of soybeans in the US from 2016–2024^9^. Approximately 75% of Illinois land area is farmland^19^. Over 30% of Illinois’ farmland (> 40,000 km^2^/10,000,000 acres) is devoted to soybeans.

2,4-D was the fourth most used pesticide by weight of active ingredient on soybeans in 2017, however second in 2023 in Illinois^20^ (behind glyphosate).

### Data

Publicly available data on pesticide application rates, the geographic distribution of crops, and the population of Illinois were used to complete this analysis.

Average state-level annual 2,4-D application rates were acquired from the United States Department of Agriculture’s Chemical Use Program which obtains data from agency-conducted farmer surveys^20^.

The area of soybeans planted per county was obtained from the National Agricultural Statistics Service (USDA-NASS). Where data was unavailable, the area was calculated from the CropScape Cropland Data Layer (USDA) raster and indicated.

Population information was obtained from the 2020 U.S. 1000 × 1000 m gridded population count dataset generated by the Socioeconomic Data and Applications Center (SEDAC) Project at Columbia University^21,22^. This dataset al.locates census block population and household information into latitude–longitude grids at the resolution of 30 arc-second (about 1000 m^2^ at the equator). By using the population count instead of the density, distortion of the pixel area is avoided. The SEDAC data was selected to avoid irregular unit shapes of census units and to achieve high-resolution exposure information that could be upscaled.

### Analysis

We performed two primary analyses. First, we compared the rate of change of 2,4-D use on the county level. Second, we used a crop-area pesticide density weighted buffer model to indicate potential exposure similar to previous studies^4,17^. All analysis and maps were created using ArcGIS Pro (Esri version 3.0).

The total area of soybeans planted was divided by the annual use of 2,4-D on soybeans to obtain an average annual pesticide application density of 2,4-D (kg/km^2^) (Table 1) for each crop year.

$${D}_{P}=\:\frac{\sum\:{F}_{i}}{{A}_{T}},$$

where $\:{D}_{P}\:$is pesticide density, $\:{F}_{i}$ is the sum (kg) of the various formulations of 2,4-D reported at the state-level, and $\:{A}_{T}$ is the total area (km^2^) of soybeans planted reported at the state-level by the USDA-NASS Agricultural Survey^23^.

**Table 1:** 2,4-D application density per year.

	2017	2020	2023
Soybean area planted (km^2^)	42,896.72	41,682.66	41,885.00
2,4-D applied (kg)	482,621.89	987,016.19	2,111,470.76
Application density (kg/km^2^)	11.25	23.68	50.41

**Fig. 1:**
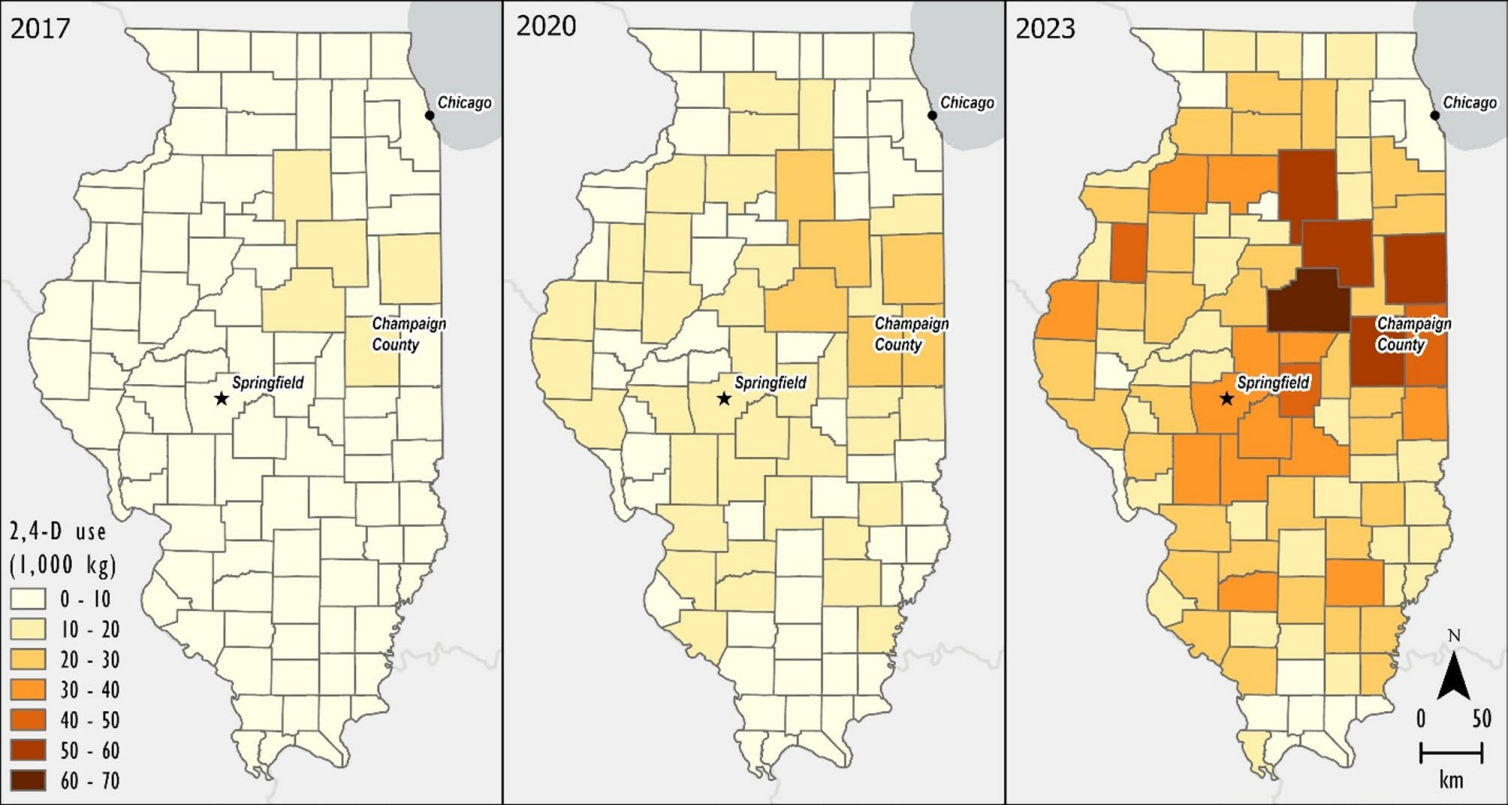
Use of 2,4-D in 1000 kg applied annually to soybeans per county. (Created in ArcPro v. 3.0 https://www.esri.com/en-us/home).

The pesticide application density was multiplied by the area of crops in each county to obtain county level 2,4-D use (kg) (Fig. 1).

$${A}_{C}\times\:\:{D}_{P}=\:{W}_{C}\:$$

where $\:{A}_{C}$ is the area of soybeans planted in each county and $\:{W}_{C}$ is the weight (kg) of applied 2,4-D (active ingredient) for each county.

**Fig. 2:**
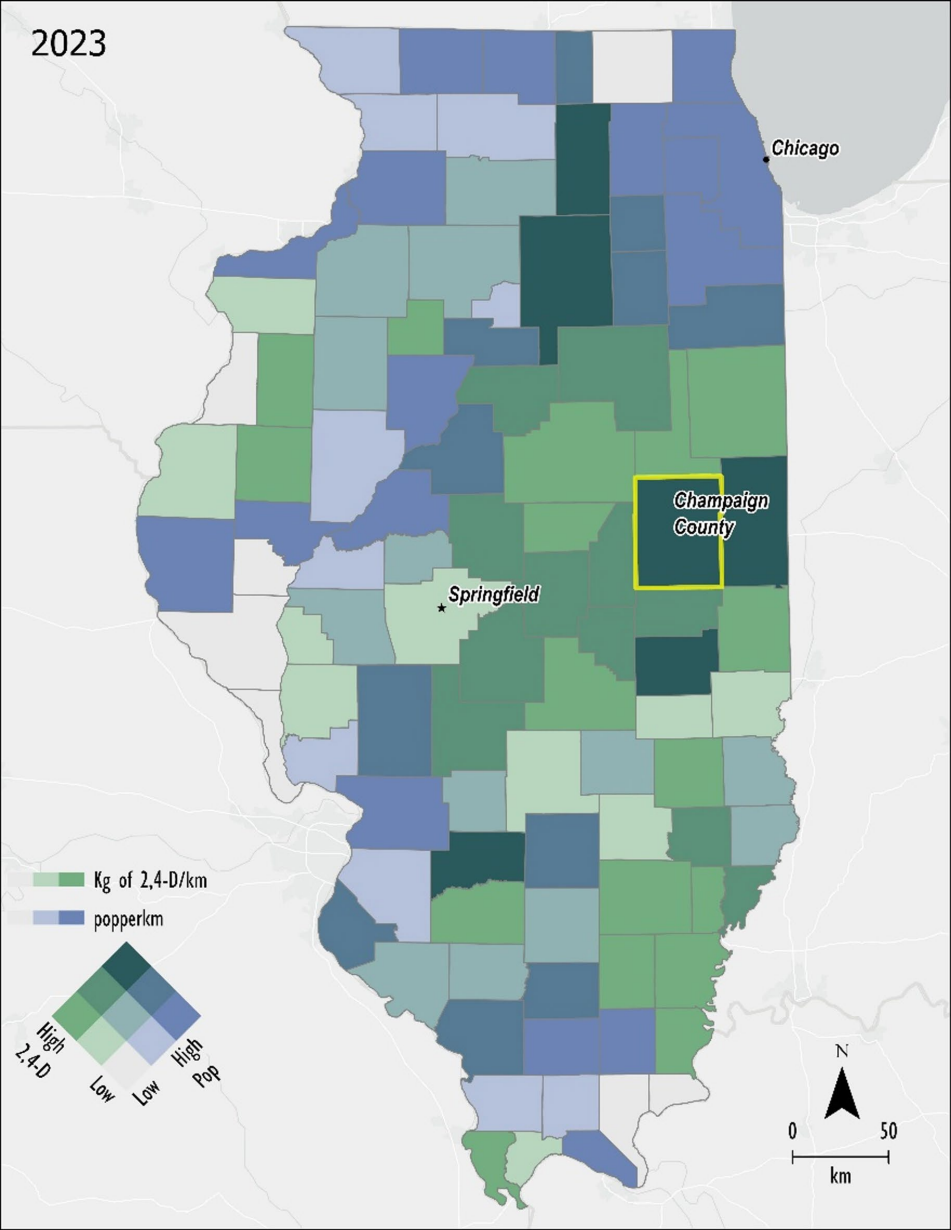
Bivariate analysis of population and 2,4-D application normalized by km^2^. Counties of concern are darkest green. Champaign County chosen for further analysis is outlined in yellow. (Created in ArcPro v. 3.0 https://www.esri.com/en-us/home).

Bivariate analysis comparing population density (population/km^2^) and 2,4-D pesticide density (kg/km^2^) was used to identify counties of concern (Fig. 2; Table 2). The outcome was comparable for all 3 years of data (2017, 2020, 2023), therefore the most recent year of data was chosen to display.

**Table 2:** Bivariate results comparing population and 2,4-D pesticide density (kg/km^2^) 2023.

County name	Population	2,4-D Density (kg/km^2^)	2,4-D Application (kg)
Champaign	206,525	20.6	53,200
LaSalle	109,495	18.0	52,900
Vermilion	74,113	18.4	42,800
DeKalb	100,686	24.2	24,900
Coles	47,076	17.7	23,400
Clinton	36,998	17.3	21,200

**Fig. 3:**
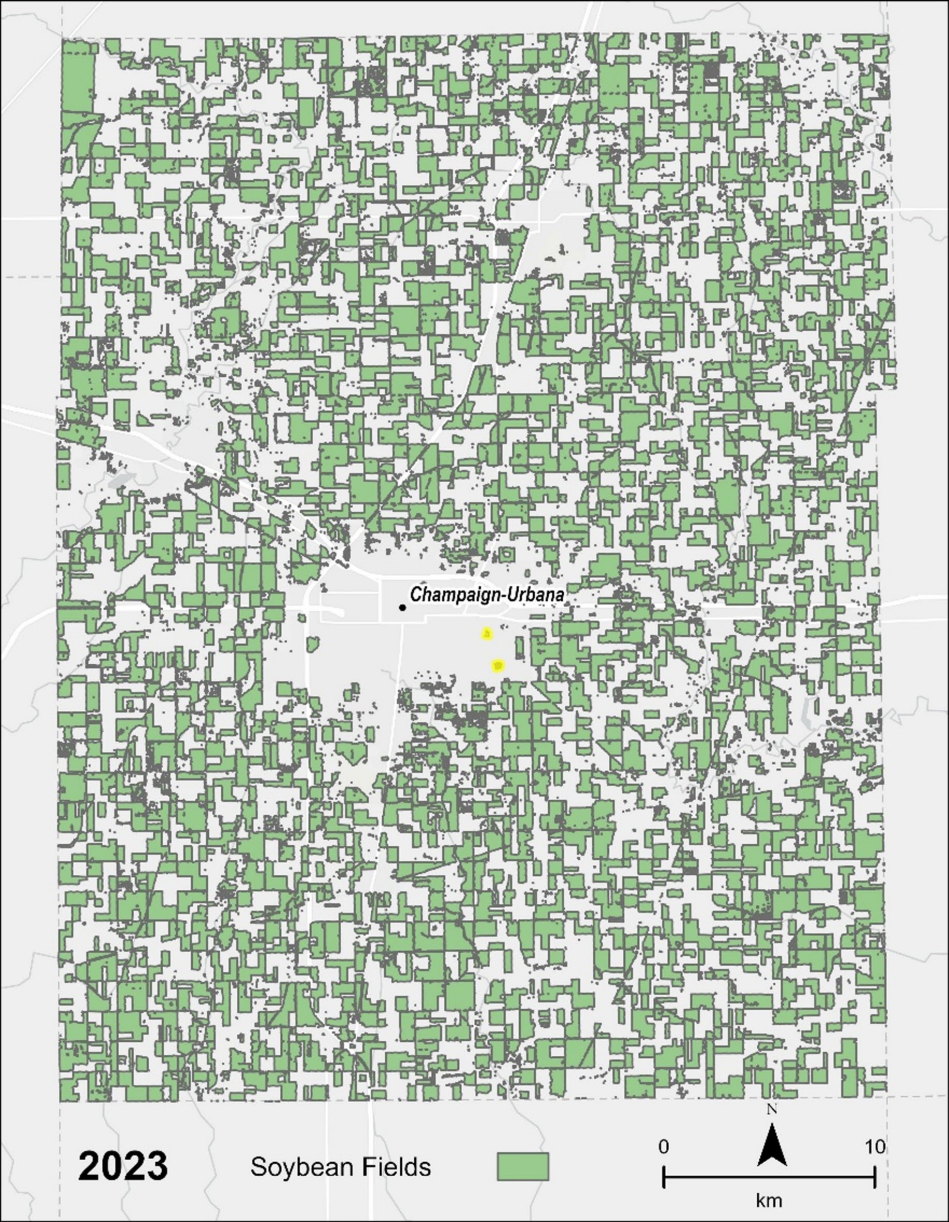
Soybean fields in Champaign County in 2023. (Created in ArcPro v. 3.0 https://www.esri.com/en-us/home).

Champaign County (Fig. 3) was ultimately selected for a more comprehensive analysis using a buffer-based exposure model. Also, according to the USDA 2022 Census of Agriculture, there are 2440 adult agricultural workers out of a population of 206,169 or 0.01% of the population. Therefore, Champaign County is an ideal example of potential non-occupational exposure.

**Fig. 4:**
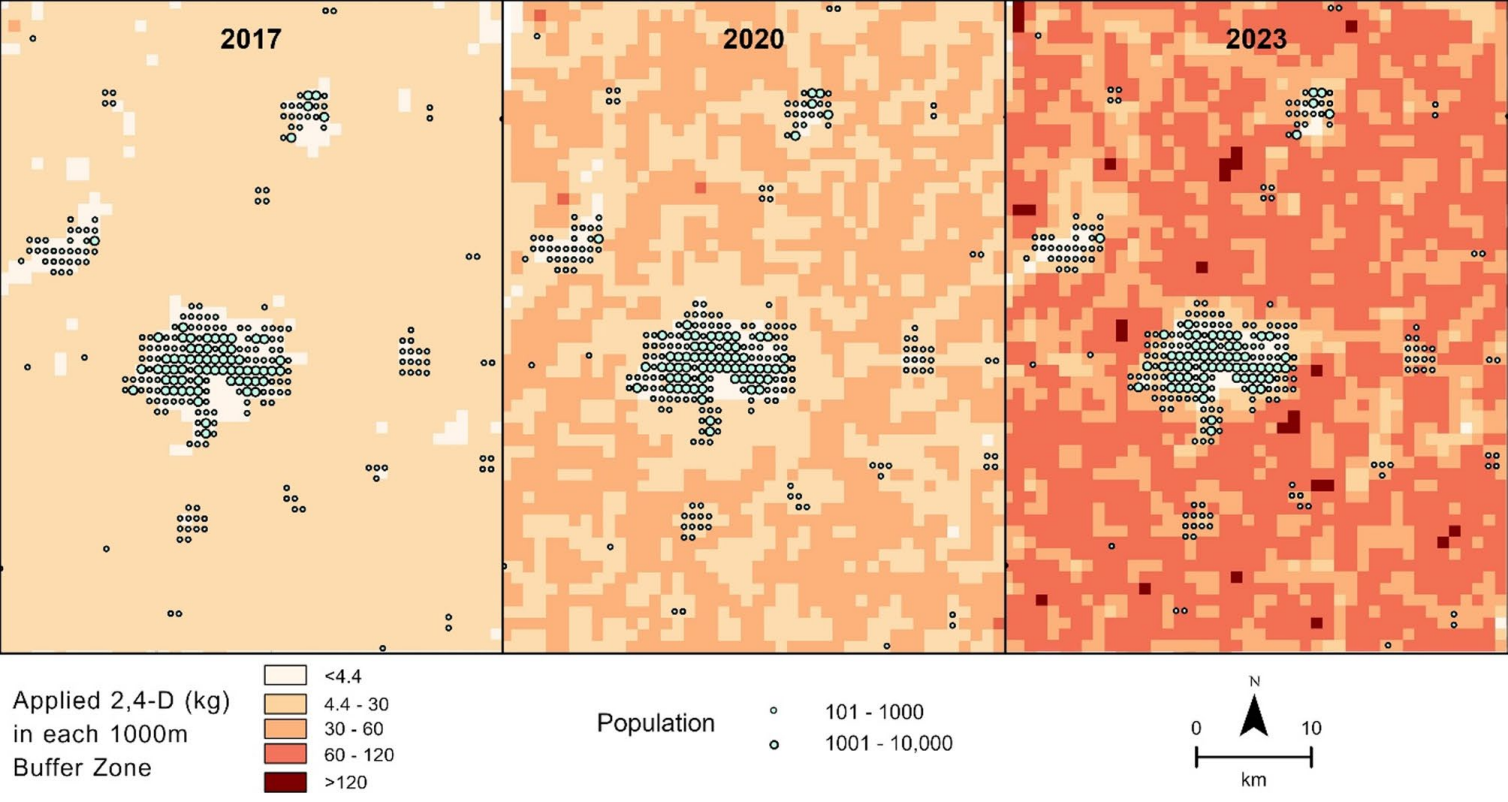
Change in 2,4-D application intensity in Champaign County 2017–2023. (Created in ArcPro v. 3.0 https://www.esri.com/en-us/home).

Exposure analysis is premised on proximity, pesticide density and weighted by area of crops within the buffer zone. This method has been described previously in the literature^4^. Briefly, a population center with a 250 m radius in each grid (~ 1 km^2^), and a donut buffer zone of 750 m creates a total buffer zone with a 1 km^5,16^ radius which encompasses the entire 1000 m x 1000 m grid. By multiplying the area of soybeans within each buffer zone by the average 2,4-D density (kg/km^2^) the total weight of applied 2,4-D (active ingredient) for each zone was obtained (Fig. 4).

$${A}_{B}\times\:\:{D}_{P}=\:{W}_{B},$$

where $\:{A}_{B}$ is the area (km^2^) of soybeans planted in the buffer zone of 1 km radius, $\:{D}_{P}$ is the 2,4-D density (kg/km^2^) and the resulting $\:{W}_{B}\:$is the total weight of applied 2,4-D.

## Results

**Fig. 5:**
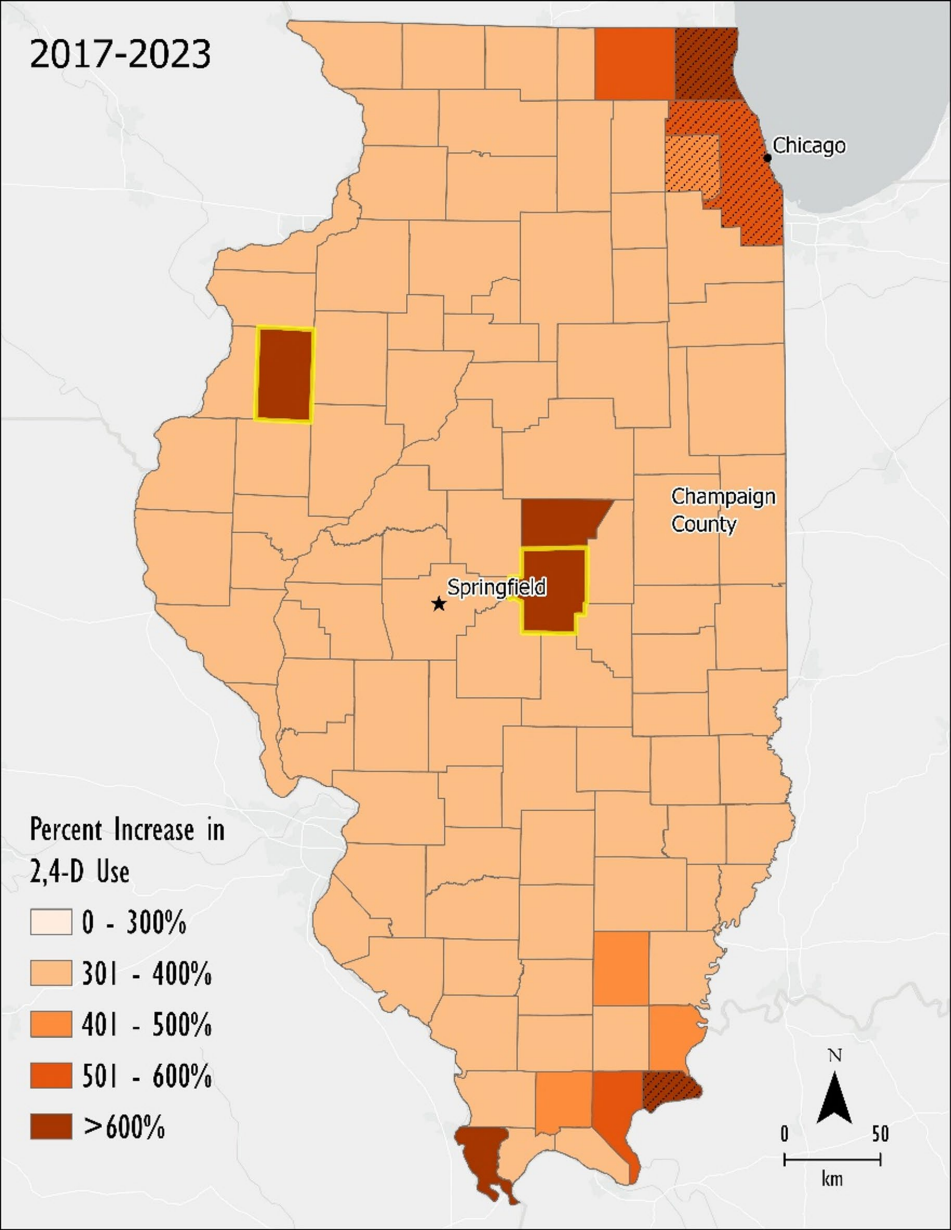
County level percent change between 2017 and 2023. Hashmarked counties had less than 40.5 km^2^ (10,000 acres) of soybeans planted in 2023. (Created in ArcPro v. 3.0 https://www.esri.com/en-us/home).

Figure 5 shows the percentage change of 2,4-D applied to soybeans in each county from 2017 to 2023 (Table 3). The counties with less than 40.5 km^2^ (10,000 acres) are indicated on the map with hashmarks. Given the scale of agriculture and soybean production in Illinois (> 40,000 km^2^/10,000,000 acres), 40.5 km^2^ planted is minimal.

**Table 3:** Rate of change of 2,4-D application and soybean area planted 2017–2023.

County	2,4-D applied to soybeans (kg)		Percent change	Soybean acres planted		Percent change
	2017	2023		2017	2023	
Adams	6281.8	28,350.7	351.3	138,000	139,000	0.7
Alexander	1565.9	12,709.8	711.7	34,400	62,315	81.1
Bond	3837.4	15,990.6	316.7	84,300	78,400	− 7.0
Boone	2098.5	9953.3	374.3	46,100	48,800	5.9
Brown	1670.6	6812.3	307.8	36,700	33,400	− 9.0
Bureau	7146.7	33,755.7	372.3	157,000	165,500	5.4
Calhoun	978.7	3977.3	306.4	21,500	19,500	− 9.3
Carroll	2144.0	9667.8	350.9	47,100	47,400	0.6
Cass	2995.2	12,196.9	307.2	65,800	59,800	− 9.1
Champaign	11,744.2	53,234.0	353.3	258,000	261,000	1.2
Christian	8330.2	34,367.5	312.6	183,000	168,500	− 7.9
Clark	4711.3	19,172.4	306.9	103,500	94,000	− 9.2
Clay	5075.5	20,396.2	301.9	111,500	100,000	− 10.3
Clinton	5052.7	21,212.0	319.8	111,000	104,000	− 6.3
Coles	5348.6	23,353.6	336.6	117,500	114,500	− 2.6
Cook	265.8	1860.6	600.0	5839	9122	56.2
Crawford	4165.1	18,132.2	335.3	91,500	88,900	− 2.8
Cumberland	3391.3	13,665.4	303.0	74,500	67,000	− 10.1
Dekalb	5712.8	24,883.3	335.6	125,500	122,000	− 2.8
Dewitt	4520.2	32,354.0	615.8	99,300	158,628	59.7
Douglas	5189.3	22,129.8	326.5	114,000	108,500	− 4.8
Dupage	91.6	540.1	489.8	2012	2648	31.6
Edgar	7624.6	31,920.0	318.6	167,500	156,500	− 6.6
Edwards	2175.9	10,238.9	370.6	47,800	50,200	5.0
Effingham	4643.1	19,233.6	314.2	102,000	94,300	− 7.5
Fayette	6896.3	28,452.7	312.6	151,500	139,500	− 7.9
Ford	6054.2	27,126.9	348.1	133,000	133,000	0.0
Franklin	3846.5	15,929.4	314.1	84,500	78,100	− 7.6
Fulton	5462.4	25,495.2	366.7	120,000	125,000	4.2
Gallatin	3892.0	21,840.9	461.2	85,500	107,084	25.2
Greene	4533.8	21,619.9	376.9	99,600	106,000	6.4
Grundy	4369.9	17,907.8	309.8	96,000	87,800	− 8.5
Hamilton	4597.5	24,750.3	438.3	101,000	121,348	20.1
Hancock	7169.4	32,531.9	353.8	157,500	159,500	1.3
Hardin	235.6	1775.4	653.4	5177	8705	68.2
Henderson	2767.6	12,135.7	338.5	60,800	59,500	− 2.1
Henry	8057.1	35,183.4	336.7	177,000	172,500	− 2.5
Iroquois	13200.8	58,944.9	346.5	290,000	289,000	− 0.3
Jackson	4410.9	21,228.3	381.3	96,900	104,080	7.4
Jasper	5348.6	22,537.8	321.4	117,500	110,500	− 6.0
Jefferson	4574.8	20,253.4	342.7	100,500	99,300	− 1.2
Jersey	2540.0	10,667.2	320.0	55,800	52,300	− 6.3
Jo Daviess	2148.6	9647.4	349.0	47,200	47,300	0.2
Johnson	1028.8	5746.8	458.6	22,600	28,176	24.7
Kane	2699.3	11,197.5	314.8	59,300	54,900	− 7.4
Kankakee	6372.8	28,146.7	341.7	140,000	138,000	− 1.4
Kendall	2936.1	12,013.3	309.2	64,500	58,900	− 8.7
Knox	5940.4	26,515.0	346.4	130,500	130,000	− 0.4
Lake	482.3	3418.0	608.7	10,595	7600	− 28.3
Lasalle	11,493.8	52,930.6	360.5	252,500	259,500	2.8
Lawrence	3823.7	16,357.7	327.8	84,000	80,200	− 4.5
Lee	5803.8	27,534.8	374.4	127,500	135,000	5.9
Livingston	13,064.3	57,721.1	341.8	287,000	283,000	− 1.4
Logan	7123.9	31,206.1	338.0	156,500	153,000	− 2.2
Macon	6281.8	45,080.4	617.6	138,000	221,024	60.2
Macoupin	7419.8	34,571.5	365.9	163,000	169,500	4.0
Madison	5462.4	22,843.7	318.2	120,000	112,000	− 6.7
Marion	4756.9	19,478.3	309.5	104,500	95,500	− 8.6
Marshall	3687.1	16,500.5	347.5	81,000	80,900	− 0.1
Mason	4374.5	18,254.6	317.3	96,100	89,500	− 6.9
Massac	1646.5	7261.0	341.0	36,171	35,600	− 1.6
McDonough	5530.7	26,107.1	372.0	121,500	128,000	5.3
Mchenry	2958.8	19,510.9	559.4	65,000	95,660	47.2
Mclean	14,179.5	61,596.4	334.4	311,500	302,000	− 3.0
Menard	3036.2	13,094.3	331.3	66,700	64,200	− 3.7
Mercer	4916.2	21,721.9	341.8	108,000	106,500	− 1.4
Monroe	3860.1	17,030.8	341.2	84,800	83,500	− 1.5
Montgomery	7123.9	32,124.0	350.9	156,500	157,500	0.6
Morgan	5667.3	24,271.4	328.3	124,500	119,000	− 4.4
Moultrie	3709.9	16,602.5	347.5	81,500	81,400	− 0.1
Ogle	5007.2	23,149.6	362.3	110,000	113,500	3.2
Peoria	3973.9	17,459.1	339.3	87,300	85,600	− 1.9
Perry	4278.9	17,805.9	316.1	94,000	87,300	− 7.1
Piatt	6008.7	24,169.5	302.2	132,000	118,500	− 10.2
Pike	5963.1	26,515.0	344.6	131,000	130,000	− 0.8
Pope	714.7	4496.4	529.2	15,700	22,045	40.4
Pulaski	2007.4	8811.1	338.9	44,100	43,200	− 2.0
Putnam	1229.0	5323.4	333.1	27,000	26,100	− 3.3
Randolph	5212.1	23,251.6	346.1	114,500	114,000	− 0.4
Richland	4165.1	16,949.2	306.9	91,500	83,100	− 9.2
Rock Island	2071.2	10,177.7	391.4	45,500	49,900	9.7
Saline	2599.2	11,299.5	334.7	57,100	55,400	− 3.0
Sangamon	7670.1	35,081.4	357.4	168,500	172,000	2.1
Schuyler	2890.5	11,952.2	313.5	63,500	58,600	− 7.7
Scott	2207.7	10,646.8	382.3	48,500	52,200	7.6
Shelby	8079.8	36,203.2	348.1	177,500	177,500	0.0
St. Clair	5143.8	22,129.8	330.2	113,000	108,500	− 4.0
Stark	3259.2	13,502.3	314.3	71,600	66,200	− 7.5
Stephenson	4074.1	16,418.9	303.0	89,500	80,500	− 10.1
Tazewell	5462.4	24,679.4	351.8	120,000	121,000	0.8
Union	1606.9	6791.9	322.7	35,300	33,300	− 5.7
Vermilion	9786.8	42,831.9	337.6	215,000	210,000	− 2.3
Wabash	2289.7	10,667.2	365.9	50,300	52,300	4.0
Warren	5485.2	42,096.6	667.5	120,500	206,395	71.3
Washington	6759.7	30,900.2	357.1	148,500	151,500	2.0
Wayne	7101.1	32,633.9	359.6	156,000	160,000	2.6
White	5849.3	24,781.3	323.7	128,500	121,500	− 5.4
Whiteside	4433.7	20,375.8	359.6	97,400	99,900	2.6
Will	5121.0	20,804.1	306.2	112,500	102,000	− 9.3
Williamson	1543.1	7301.8	373.2	33,900	35,800	5.6
Winnebago	2494.5	10973.1	339.9	54,800	53,800	− 1.8
Woodford	5599.0	23,761.5	324.4	123,000	116,500	− 5.3

The median rate of change was an increase of 341%. The county with the smallest rate of change had a 302% increase of 2,4-D application between 2017 and 2023, while the largest was a 712% increase. Two counties, Warren and Macon, had among the largest increases in 2,4-D use also had large increases in the area of soybeans planted and are in the top 10 counties in the state in 2023 of the most km^2^ of soybeans planted (outlined on Fig. 5). Warren County had a 667% increase in 2,4-D use and a 71% increase in the area of soybeans planted. Macon County had a 617% increase in 2,4-D use and a 60% increase in the area of soybeans planted.

Using the population grid center as the approximate population location it was found that 98.9–99.7% of the total population of Champaign County lived within 1 km of at least 0.04 km^2^ (10 acres) of soybean crops from 2017 to 2023.

**Fig. 6:**
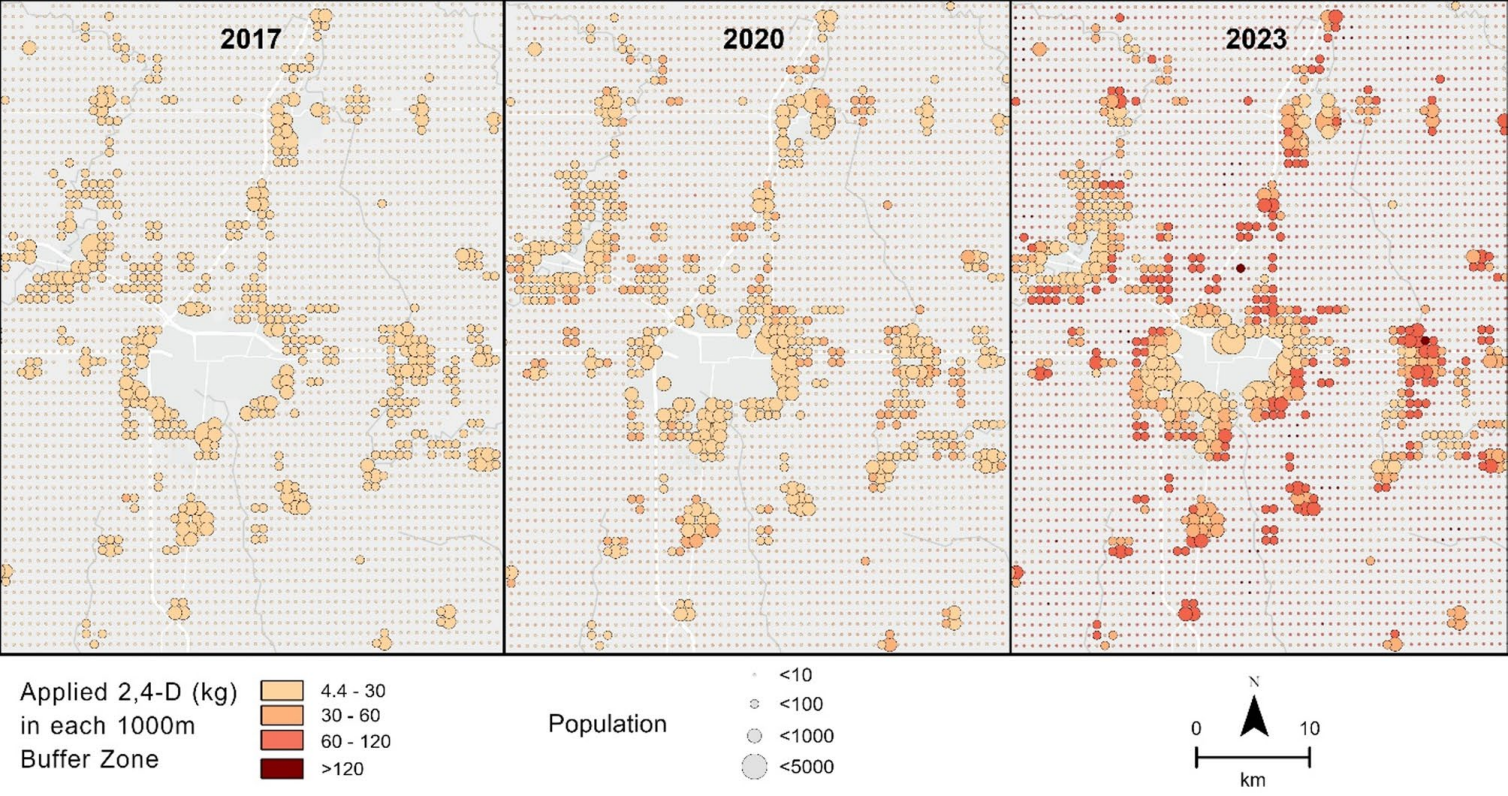
Magnitude of population and 2,4-D intensity change in Champaign County 2017–2023. (Buffer zones below 4.4 kg threshold were removed for clarity.) (Created in ArcPro v. 3.0 https://www.esri.com/en-us/home).

Application of 4.4 kg of 2,4-D within a 1 km buffer zone has been found to be associated with a greater than 100% increase in concentration of indoor dust contamination^16^. Using this threshold, 24.5% (~ 57,000 people) of the population was potentially exposed in 2017, this increased to 44.5% (~ 103,000 people) of the population in 2023 as shown in Fig. 6.

In 2017, the two buffer zones where the most 2,4-D (30 kg) was applied to soybean crops represented 0.01% (14 people) of the population potentially exposed. Using 30 kg as a reference point, 4.0% of the population were at potential risk of this level of exposure in 2020. In 2023, 20.2% (~ 47,000 people), which translates to a > 330,000% increase from 2017, of the population were at potential risk of this level of exposure. (Fig. 6)

## Discussion

**Fig. 7:**
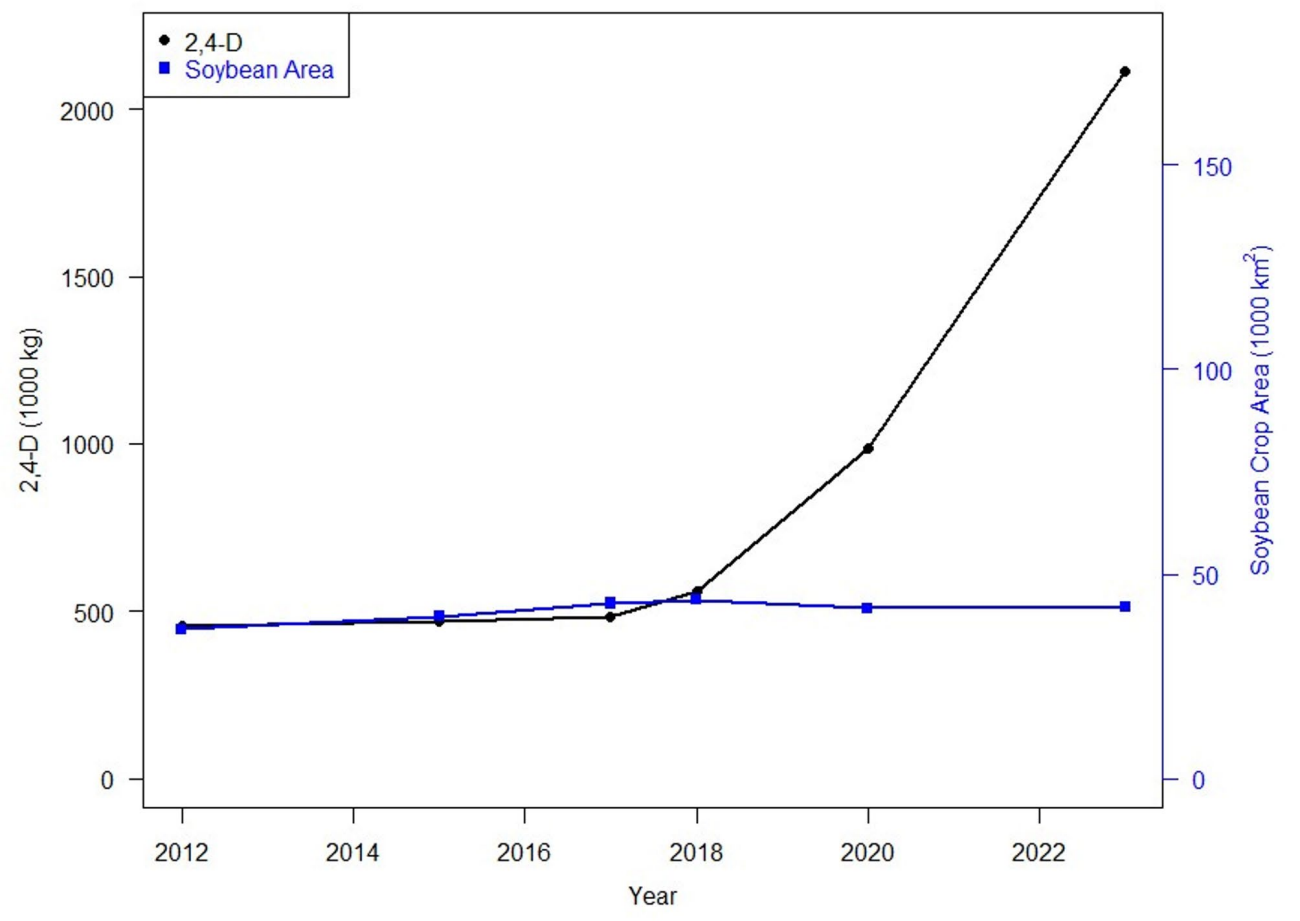
2,4-D application on soybeans and soybean area 2017–2023. Y1 and Y2 axes are proportional.

This study uses a GIS pesticide density, crop area buffer model to show spatially increasing use of 2,4-D over time and the potentially at-risk exposed nearby population. From 2017 to 2023 agricultural use of 2,4-D applied to soybeans in Illinois increased over 1.5 million kg (Fig. 7). Bivariate analysis correlating population and 2,4-D use identified Champaign County for further analysis. Using a crop-weighted pesticide density buffer geospatial model with a 4.4 kg threshold we found from 2017 to 2023 we found the population at risk of exposure increases from 24.5 to 44.5%. Additionally, in 2017 30 kg applied per 1000 m buffer zone was the maximum 2,4-D application potentially exposing 0.01% (~ 14 people) of the population, in 2023 the population exposed to this reference level had increased to 20.2% of the population.

The agricultural use of the herbicide 2,4-D has increased exponentially since 2017, while the area of soybeans planted has remained relatively stable over time^9^.

Weed resistance due to the intensive use of glyphosate has led to the increasing use of older herbicides like dicamba and 2,4-D with less favorable physical characteristics such as drift and volatility^24^. Novel herbicide resistant-soybean crops have come on the market and resulted in a dramatic increase in 2,4-D use over time. Being able to specify where potential non-occupational exposure is occurring is valuable.

Pesticide application densities (kg/km^2^) are strong predictors of indoor dust concentrations for 2,4-D^16^. It has previously been found that increasing area of corn and soybean fields within 750 m of homes are significantly associated with the probability of detecting 2,4-D and increasing concentrations of 2,4-D in dust in homes. The same study, based in Iowa, detected 2,4-D in the dust of 95% of the homes (*n* = 112)^5^. These associations remained after accounting for whether residents were agricultural workers or not.

Carpet dust is an effective indicator of long-term chemical exposure in homes, as it acts as a reservoir for pesticides and other chemicals, protected from degradation by sunlight, moisture, and microorganisms^5,25^.

Madrigal et al. found in 2024 that increased density of pesticide agricultural applications led to a 60–100+% increase in 2,4-D concentration in dust within a 1 km buffer of residences compared to homes that did not have agriculture within the buffer zone. 2,4-D was detected in the dust of > 97% of homes in California (*n* = 578) irrespective of nearby agricultural land^16^. This study also found that wind direction did not substantially change the results.

2,4-D was detected in > 80% of households in North Carolina and Ohio (*n* = 135)^26^.

Data from 2007 to 2014 of the National Health and Nutrition Examination Study (NHANES) conducted by the CDC, has shown that increasing agricultural use of 2,4-D has been significantly correlated with increased 2,4-D biomarker detection in the population^27^. The most recent cycle of NHANES testing for 2,4-D is 2015–2016, which is before this recent increase in use, detected 2,4-D in 85% of children^28^.

Exposure to 2,4-D has been linked to a range of adverse health outcomes, including endocrine disruption, reproductive disorders, genetic alterations^29^, immunosuppression and carcinogenic effects. The herbicide has been shown to disrupt endogenous hormone production and function. Specifically testosterone, therefore negatively affecting semen quality^6,30–32^, and there is also evidence of thyroid disruption^33^. This has led to its categorization as a potential endocrine-disrupting chemical (EDC).

Associations between 2,4-D exposure and increased birth defects and adverse birth outcomes have been reported^34–36^. In 2023, a study found an association between urinary detection of 2,4-D and lower scores for neurobehavioral performance^37^. Another study found neurotoxicity associated with chronic exposure exposed to environmental levels of 2,4-D in rat models^38^.

Environmental exposure to 2,4-D has been implicated in exacerbating antibiotic resistance in potential human or animal pathogens^39^. It has also been implicated in immune suppression in in vivo models^40^.

Substantial research has been done to determine the cancer potency of 2,4-D. In 2015 (updated in 2019), the International Agency for Reseaerch on Cancer (IARC) first classified 2,4-D as a possible human carcinogen (Group 2B). This finding was based on limited evidence in humans and sufficient evidence in animals. A recent meta-analysis concluded that higher exposure to 2,4-D is associated with an increased risk of non-Hodgkins lymphoma^41^. Additionally, a 2024 study found chronic exposure to 2,4-D in women has been associated with an increased risk of breast cancer diagnosis and metastasis development^42^.

One of the challenges of this study is setting metrics. While the current scale of use of 2,4-D is unprecedented, it is repeating history and is a consequence of the “pesticide treadmill”. This is an on-going cycle where farmers continually increase their pesticide use to compensate for weed resistance from a lack of new weed management technology^43^. For example, glyphosate resistant GE crops became commercially available in 1996, by 2012 glyphosate agricultural use had increased > 900%, with soybeans accounting for about half of the use^44,45^. As of the writing of this paper, there are 373 glyphosate resistant weeds and 50 weeds resistant to 2,4-D^46^.

### Strengths

There are numerous strengths to this study. Few studies have attempted to model potential population exposure at such a high spatial resolution^47^. Also, as noted above, Champaign County is an excellent example to use for non-occupational exposure. While being in the midst of so much agricultural area, the primary employer is education and education related fields due to two universities in the counties, which employs almost 17% of the adult population^48^.

By using publicly available data sets, this method is broadly generalizable to multiple crops, chemicals and geographical areas. By using the 1000 m x1000m gridded population data, the method provides a higher spatial resolution than using U.S. Census data. County level crop area data provided by NASS is one of the most reliable sources in the US (user accuracy 95–97%)^49,50^. Public health data is usually on a larger scale to protect privacy, the results of this model can also be upscaled as needed.

This buffer-based model is also flexible. There is a gap in the literature using different buffer distances^51^, and while we have also only examined one buffer distance here, the model is easily modifiable to other distances.

To our knowledge, this is the first time population data is incorporated with the spatiotemporal trends of 2,4-D use. This is also the first time that changes in herbicide use and potential exposure has been mapped.

### Limitations

While GIS is a powerful tool for estimating pesticide exposure, it is important to acknowledge its limitations. Exposure estimates using GIS are rough approximations, as available data often lack the precision needed for more accurate calculations. While proximity has been used as a surrogate for potential exposure, no individuals have had their exposure measured in this study. Exposure misclassification is possible from satellite-based datasets because of errors in geocoding^3,52^. CropScape, the crop raster layer data, results in an underestimation bias when pixel counting^53^. This type of model relies on several assumptions. By assigning pesticide use per area, this assumes an even distribution and spread.

This study assumes non-occupational exposure due to proximity to 2,4-D agricultural use, but due to the ubiquitous use of 2,4-D residentially and on other crops, exposure from other routes is also possible and even likely. Therefore, assuming exposure risk just from this study is a potential underestimation.

## Conclusions

In this ecological study we employ a GIS model that uses 2,4-D applied to soybeans in Illinois as a case study to show recent spatiotemporal trends and the potential non-occupational population exposure risk. 2,4-D use has increased an average of 363.2% from 2017 to 2023 over all the counties in Illinois. Using an area-weighted, pesticide density 1 km buffer model with a 1000 × 1000 m gridded population data set, we found 44.5% of the population of Champaign County was potentially exposed to a 4.4 kg of 2,4 D or higher threshold in 2023 vs. 14.5% of the population in 2017.

Non-occupational exposure is difficult to determine. Biomonitoring is the gold standard which is both invasive and expensive. Environmental monitoring can be technically difficult. Using this type of model can identify precisely where to focus efforts and resources for epidemiological and environmental studies which can help address growing concerns about the environmental and public health impacts of modern agriculture. The identification of specific locations where exposure potential is high allows for the possibility of targeted individual-level interventions.

This type of data can be used to identify at-risk populations or in conjunction with public health data to identify potential associations with health consequences. This model can be a template that can be applied and expanded to other pesticides, crops and geographic regions. Estimating potential 2,4-D non-occupational exposure at the county level for the state of Illinois due to proximity to soybean crops as well as changes over time shows the relevance of this method and its generalizability.

## Data Availability

The raw data that support the findings in this paper are openly available at https://quickstats.nass.usda.gov/ (United States Department of Agriculture- National Agriculture Statistics Service), https://nass.usda.gov/Research_and_Science/Cropland/Release/index.php (United States Department of Agriculture Cropland Data Layer), https://www.nass.usda.gov/Surveys/Guide_to_NASS_Surveys/Chemical_Use/ (United States Department of Agriculture Chemical Use Survey). Population data is available from https://cmr.earthdata.nasa.gov/search/concepts/C1597158029-SEDAC.html (National Aeronautics and Space Administration) and https://data.census.gov/ (United States Census Bureau).

## Abbreviations

2,4-D: 2,4-Dichlorophenoxyacetic acid
GE: Genetically engineered
GIS: Geographic information systems
USDA: United States Department of Agriculture
NASS: National Agricultural Statistics Service
